# Similarities and differences among the chromosomes of the wild guinea pig *Cavia tschudii* and the domestic guinea pig *Cavia porcellus* (Rodentia, Caviidae)

**DOI:** 10.3897/CompCytogen.v8i2.7509

**Published:** 2014-07-14

**Authors:** Laura I. Walker, Miguel A. Soto, Ángel E. Spotorno

**Affiliations:** 1 Laboratorio de Citogenética Evolutiva, Instituto de Ciencias Biomédicas, Facultad de Medicina, Universidad de Chile, Santiago, Chile. Casilla 70061, Santiago 7, Chile

**Keywords:** Karyotype, G, C and AgNOR banding, active NORs, pericentric inversions, domestication

## Abstract

*Cavia tschudii* Fitzinger, 1867 is a wild guinea pig species living in South America that according to the analysis of mitochondrial genes is the closest wild form of the domestic guinea pig. To investigate the genetic divergence between the wild and domestic species of guinea pigs from a cytogenetic perspective, we characterized and compared the C, G and AgNOR banded karyotypes of molecularly identified *Cavia tschudii* and *Cavia porcellus* Linnaeus, 1758 specimens for the first time. Both species showed 64 chromosomes of similar morphology, although *C. tschudii* had four medium size submetacentric pairs that were not observed in the *C. porcellus* karyotype. Differences in the C bands size and the mean number of AgNOR bands between the karyotypes of the two species were detected. Most of the two species chromosomes showed total G band correspondence, suggesting that they probably represent large syntenic blocks conserved over time. Partial G band correspondence detected among the four submetacentric chromosomes present only in the *C. tschudii* karyotype and their subtelocentric homologues in *C. porcellus* may be explained by the occurrence of four pericentric inversions that probably emerged and were fixed in the *C. tschudii* populations under domestication. The role of the chromosomal and genomic differences in the divergence of these two *Cavia* species is discussed.

## Introduction

*Cavia tschudii* Fitzinger, 1867 is a wild species of guinea pig (Rodentia, Caviidae) which inhabits northern Chile, southern Peru and Bolivia and northwestern Argentina (Weir 1974, Woods and Kilpatrick 2005). The domestic guinea pig *Cavia porcellus* Linnaeus, 1758 has a cosmopolitan distribution and is an experimental animal, pet, and even is consumed as food in countries of the Andean Altiplano (Tello 1972).

There is a consensus that *Cavia porcellus* is a domestic form derived from one of the five currently recognized wild species of guinea pigs that inhabit South America (Woods and Kilpatrick 2005). The crosses between *Cavia porcellus* and *Cavia fulgida* Wagler, 1831 yielded offspring which behaved according to the Haldane’s rule (Haldane 1922), since females were fertile and males were sterile (Detletfsen 1914). By contrast, the crosses between *Cavia porcellus* and *Cavia aperea* *sensu* Erxleben, 1777 (Pictet and Ferrero 1951, Rood 1972) and between *Cavia porcellus* and *Cavia cutleri* Tschudi, 1844 (*sensu* Bennet, 1836) (Castle 1916) produced hybrids which were fertile in both sexes*. C. aperea* or *Cavia tschudii* have been repeatedly considered as the most probable ancestor of the domestic guinea pig. Later, molecular analyses of the mitochondrial cytochrome *b* and 12S RNA genes clearly showed that the closest species to *Cavia porcellus* is *Cavia tschudii* and not the genetically related *Cavia aperea* (Spotorno et al. 2004, Dunnum and Salazar-Bravo 2010). Based on these molecular results and on the analysis of mummified guinea pig remains found in archeological sites, Spotorno et al. (2007) suggested that the domestication of the wild guinea pig occurred in southern Peru-northern Chile.

Considering that the karyotype provides useful characters in taxonomic and systematic studies and that changes in the number and structure of chromosomes may contribute to speciation (King 1993, Searle 1993, Capanna and Redi 1994, Capanna and Castiglia 2004, Marques-Bonet and Navarro 2005, Faria and Navarro 2010), we describe and compare now for the first time the G, C and AgNOR banded karyotypes in molecularly identified specimens of the wild montane guinea pig *Cavia tschudii* and the domestic guinea pig *Cavia porcellus*. Our objective is to discover the chromosomal and genomic differences between these two species of *Cavia* in relation to the divergence associated with the domestication process.

## Material and methods

Skulls, skins and liver samples for DNA analysis of all the studied animals were preserved in the collection of the Laboratorio de Citogénetica de Mamíferos, Facultad de Medicina, Universidad de Chile (LCM). We examined five *Cavia tschudii* specimens, four males (LCM 3199b, 3110, 3080, 3225) and one female (LCM 3232), collected in the locality of Molinos, Valle de Lluta, 18°23’S, 69°45’W, Arica, I Región, Chile, and four *Cavia porcellus* animals, two males (LCM 2454, 3192) from the laboratory Pirbright breed, Instituto de Salud Pública, Santiago, Chile, and two females (LCM 2479, 2489) from the Andean creole breed, Arica Agromarket, Arica, Chile.

**Figure 1.**
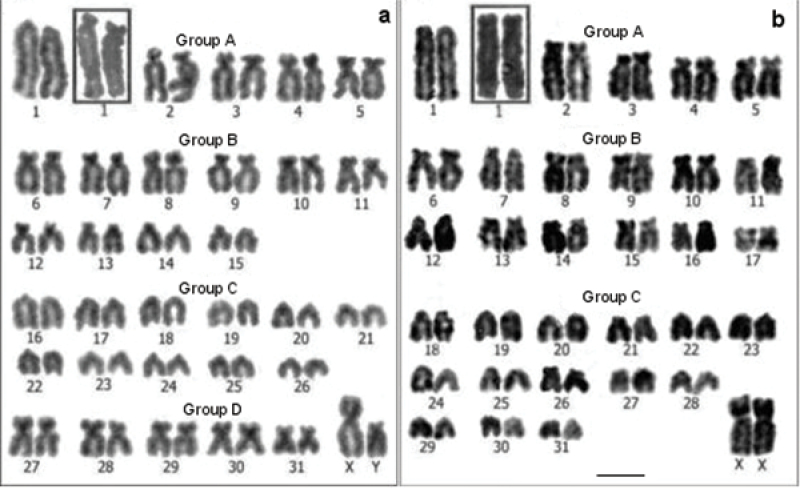
Conventional stained karyotypes: **a** *Cavia tschudii* male **b** *Cavia porcellus* female. Chromosomal pair Nº 1 shows subtelocentric morphology in other individuals of both species (**a** and **b** insets). Bar = 5 µm.

Chromosomes were obtained from marrow cells using conventional in vivo colchicine, hypotonic method, preceded by yeast injection to improve the mitotic index (Lee and Elder 1980). Metaphase cells were G-banded and C-banded by the methods described by Chiarelli et al. (1972) and Sumner (1972), respectively. The active nucleolar organizing regions (NORs) were detected by the silver staining procedure (Sánchez-Rufas et al. 1982). At least 10 good-quality metaphases for each of the staining methods per taxon were selected under a light microscope and digitally captured and stored. Chromosomes were counted, cut out and ordered by size and form using ADOBE PHOTOSHOP version 6.0. The centromeric indexes calculated by measuring the chromosomal arms in 12 metaphases of each species, allowed the classification of the chromosomes as metacentric, submetacentric, subtelocentric and telocentric (Levan et al. 1964). Chromosomes of both species were ordered in the groups defined by Fernández and Spotorno (1968) for *Cavia porcellus* (groups A, B and C), adding a fourth group (group D) of submetacentric chromosomes for *Cavia tschudii* (Fig. 1). Male and female G-banded karyotypes from each species were compared and the chromosomes were classified as having totally corresponding, partially corresponding or unique G band patterns (Spotorno 1977, Walker et al. 1979). The size and distribution of the C and AgNOR bands were evaluated in six metaphases of *Cavia tschudii* and nine of *Cavia porcellus*. To determine the total number of active AgNOR in each of the species, AgNOR^+^ sites were identified and counted in the chromosomes of 24 metaphases per species. The statistical significance of the differences was estimated using a Chi squared test.

## Results

### Chromosome number, size and morphology

For *Cavia tschudii* and *Cavia porcellus* we consistently found a 2n = 64, FNa = 100-102; the variation in the FNa of both species was due to the polymorphism of chromosome 1 (Fig. 1). *Cavia tschudii* showed five pairs of submetacentric chromosomes (group D, Fig. 1a) of which four pairs (numbers 27, 28, 29 and 30) were not present in the *Cavia porcellus* karyotype (Fig. 1b). The X chromosome of *Cavia tschudii* was a large submetacentric similar to that of *Cavia porcellus* and the Y chromosome was a subtelocentric larger than that of *Cavia porcellus* (Fig. 1).

### G bands

**Figure 2.**
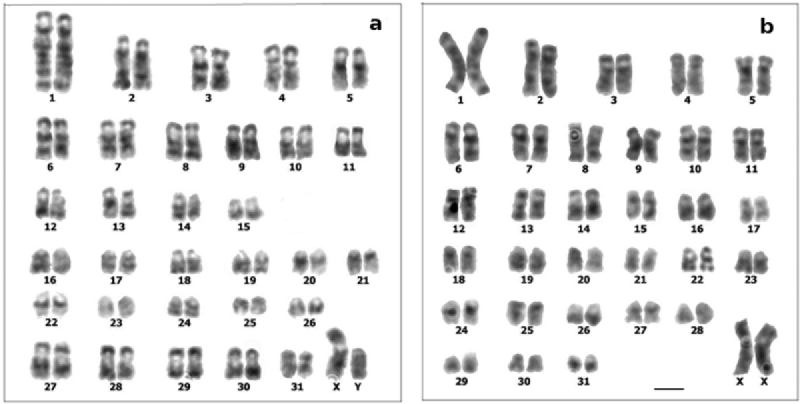
G-banded karyotypes: **a** *Cavia tschudii* male **b** *Cavia porcellus* female. Chromosomes numbered according to original karyotype descriptions (see Fig. 1). Bar = 5 µm.

**Figure 3.**
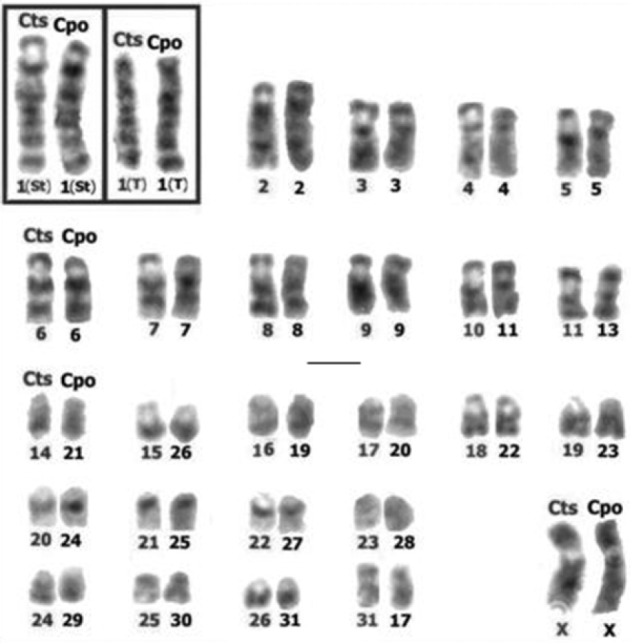
Chromosomes of *Cavia tschudii* (Cts) and *Cavia porcellus* (Cpo) with total G band correspondence. Cts chromosomes are at the left and Cpo at the right of each chromosomal group. Note that the long arms of subtelocentric (St) and telocentric (T) forms of pair 1 show total G band correspondence (inset). Chromosomes numbered according to original karyotype descriptions (see Fig. 1). Bar = 5 µm.

The comparison of *Cavia tschudii* and *Cavia porcellus* G-banded karyotypes revealed total correspondence for 25 of the 31 autosomal pairs and for the X chromosomes of both species (Figs 2, 3, Table 1). The four submetacentric chromosomes present only in the *Cavia tschudii* karyotype showed partial G band correspondence with four *Cavia porcellus* subtelocentric chromosomes (Fig. 6, Table 1). Only the Y chromosomes and two autosomal pairs (*Cavia tschudii* chromosomes 12, 13 and *Cavia porcellus* chromosomes 14, 18) were unique of each species karyotype (Table 1).

**Table 1.** Correspondence of *Cavia tschudii* and *Cavia porcellus* chromosomes according to their G band patterns ^1^.

Chromosomes with total G band correspondence																										
**Cts**	1	2	3	4	5	6	7	8	9	10	11	14	15	16	17	18	19	20	21	22	23	24	25	26	31	X
**Cpo**	1	2	3	4	5	6	7	8	9	11	13	21	26	19	20	22	23	24	25	27	28	29	30	31	17	X
Chromosomes with partial G band correspondence																										
**Cts**	27	28	29	30																						
**Cpo**	10	12	15	16																						
Unique species chromosomes																										
**Cts**	12	13			Y																					
**Cpo**			14	18		Y																				

^1^ Chromosome numbers are the one of each species karyotype (see Fig. 1); in the same column chromosomes with total or partial G band correspondence. Cts = *Cavia tschudii*, Cpo = *Cavia porcellus*.

### C bands

**Figure 4.**
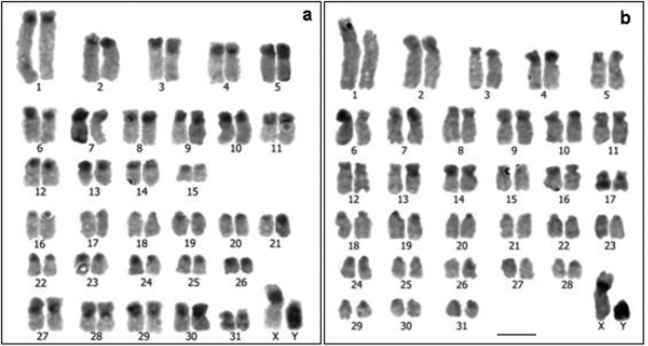
C-banded karyotypes: **a** *Cavia tschudii* male **b** *Cavia porcellus* male, showing heteromorphism for chromosome 1. Most of the chromosomes of both species were tentatively identified according size and morphology. Bar = 5 µm.

The chromosomal distribution of the C bands was similar in the karyotypes of the two species, being located preferentially in the centromeres and the short arms of the chromosomes (Fig. 4). However, the amount of constitutive heterochromatin was appreciably greater in *Cavia tschudii* than in *Cavia porcellus*, spreading over most of the short arms in several subtelocentric chromosomes (Fig. 4a). The X chromosomes of both karyotypes, equal in size, morphology and G bands (Figs 1–3), showed a C^+^ band in the paracentromeric region of the short arm (Fig. 4). Both Y chromosomes were completely heterochromatic, being larger the Y chromosome of *Cavia tschudii* than the *Cavia porcellus* one (Fig. 4).

### AgNOR bands

**Figure 5.**
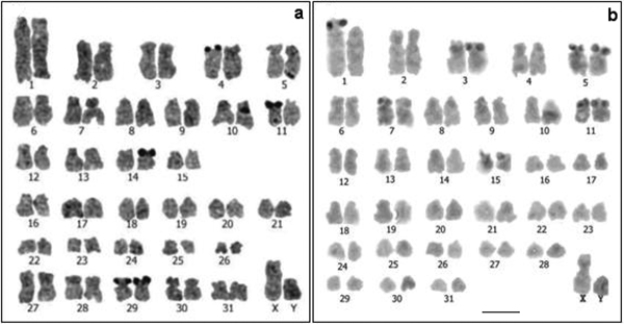
AgNOR-banded karyotypes: **a** *Cavia tschudii* male with four nucleolar chromosomal pairs (4, 11, 14 and 29) **b** *Cavia porcellus* male with five nucleolar chromosomal pairs (1, 3, 5, 7 and 11). The nucleolar chromosomes of both species were tentatively identified according to their size and morphology. Bar = 5 µm.

Multiple AgNOR bands were detected in the karyotypes of both species, consistently located in the telomeres of several chromosomal pairs (Fig. 5). The analysis of some AgNOR banded metaphases per species indicated that the number of AgNOR bands was different between the two species and also among the individuals. Thus, the results showed that in *Cavia porcellus* the mean and maximum numbers of chromosomes with active NORs (5.76 and 9.0, respectively) were higher than those of *Cavia tschudii* (4.13 and 7.0, respectively). Moreover, when we examined all the 3.072 chromosomes from 48 metaphases of both species, each of them having 64 chromosomes, we found a total of 237 AgNOR^+^ sites, 138 of them located in *Cavia porcellus* chromosomes and 99 in *Cavia tschudii* chromosomes. Accordingly, the number of chromosomes bearing active NOR was significantly higher in the *Cavia porcellus* karyotype than in the *Cavia tschudii* one (χ*^2^* = 6.956; p < 0.05; df = 1).

## Discussion

*Cavia tschudii* and *Cavia porcellus* diploid numbers (2n = 64), previously described with basic cytogenetic techniques (Ohno et al. 1961, Fernández and Spotorno 1968, Dunnum and Salazar-Bravo 2006), were confirmed; nevertheless the fundamental number of autosomal arms (FNa = 100-102) were different to those reported before. For *Cavia porcellus*, Fernández and Spotorno (1968) described an FNa = 96, while for *Cavia tschudii*, Dunnum and Salazar-Bravo (2006) found an individual in the Bolivian Altiplano with an FNa which ranged from 104 to 108. The FNa variability of guinea pig species may be due to polymorphisms for the presence of short arms in the chromosomes described as subtelocentric or telocentric in these species. The polymorphism for chromosome 1 short arms detected previously for *Cavia cobaya* Pallas, 1766, a synonym of *Cavia porcellus*, (Ohno et al. 1961, Schmid 1965, Zenzes et al. 1977) and for both species in this study, as well as the report of entirely heterochromatic short arms in the subtelocentric autosomes of *Cavia porcellus* (Bianchi and Ayres 1971), give support to such hypothesis.

The number and morphology of *Cavia tschudii* and *Cavia porcellus* chromosomes were similar to those reported for other subspecies and species of the genus *Cavia*. So, with the exception of *Cavia intermedia* Cherem, Olimpio, Ximenez, 1999, and a population of *Cavia magna* Ximenez, 1980, having 2n = 62 (Gava et al. 1998, Cherem et al. 1999, Gava et al. 2012), for all of the other taxa of the genus the same 2n = 64 diploid number has been described, although with different numbers of autosomal arms. An FNa = 124 was recorded for *Cavia aperea pamparum* (George et al. 1972); FNa = 116 for *Cavia aperea aperea* specimens from Pernambuco, Brasil (Maia 1984) and 114 for some individuals from the Bolivian lowlands (Dunnum and Salazar-Bravo 2006); FNa = 124 for *Cavia magna* and *Cavia fulgida* (Pantaleão 1978) and 114 for *Cavia nana* Thomas, 1917 (*Cavia tschudii sodalis*, 1926) (Dunnum and Salazar-Bravo 2006).

The analysis of the C bands showed that although they had a similar distribution in the chromosomes of the two species, they were smaller in size in the autosomes and in the Y chromosome of *Cavia porcellus* than in the *Cavia tschudii* ones, suggesting that a loss of heterochromatin occurred during the domestication process. In accordance with this result, measurements of the genome sizes of 31 hystricognath rodent species (Gallardo et al. 2003) indicated that the genome of *Cavia tschudii* (9.1 pg) is larger than that of *Cavia porcellus* (8.2 ± 0.4 pg), having the first species the largest genome size among the 30 diploid species analyzed.

Five chromosomal pairs bearing NOR at the short arm telomeres were found by Zenzes et al. (1977) in the karyotype of the domestic *Cavia*, so being in agreement with our results. Using a double-staining procedure they could identify those chromosomes as numbers 1, 3, 9, 12 and 14 of the quinacrine banded stained karyotype. An accurate identification of the *Cavia porcellus* and *Cavia tschudii* nucleolar chromosomes described here would require the use of a similar double-staining procedure to allow the comparisons with other descriptions.

The differences in the number of AgNOR bands found between the two *Cavia* species analyzed here and among the individuals in each of them, confirmed the tendency to variability in NOR expression usually described for mammals. It has been proposed that this variability would depend mainly on the specific metabolic demands of cells and individuals (Mikelsaar et al. 1977, Mayr et al. 1987, Sánchez et al. 1989, Suzuki et al. 1990, Berríos et al. 1992, Zurita et al. 1997, Walker et al. 1999, Walker and Flores 2007). The greater number of AgNOR bands found in the *Cavia porcellus* karyotype than in the *Cavia tschudii* one would reveal a greater transcriptional activity of the ribosomal genes in the genome of the domestic form. One possible functional explanation of this result is that since *Cavia porcellus* has been selected for productive purposes, it would require higher rates of protein synthesis than the wild form. It should be mentioned that in a recent comparison of brain gene expression levels between four pairs of domestic/wild mammals, the largest differences were found between the domestic and wild guinea pigs, although *Cavia aperea*, genetically related to *Cavia tschudii*, was used as the wild guinea pig species (Albert et al. 2012).

**Figure 6.**
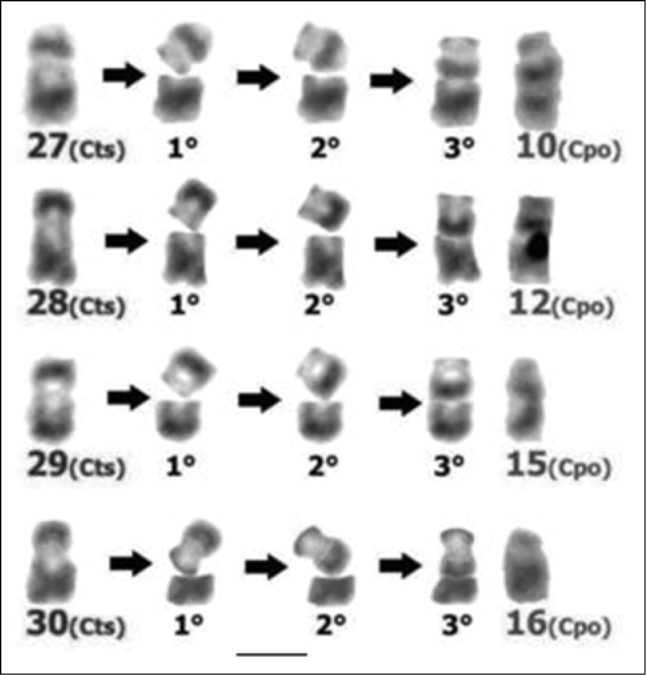
Rough simulation of the changes associated with the occurrence of pericentric inversions in *Cavia tschudii* chromosomes. Submetacentric *Cavia tschudii* chromosomes (Cts, first column at the left) that originate the subtelocentric *Cavia porcellus* chromosomes (Cpo, last column at the right): 1°) chromosomal break, 2°) rotation of the cleaved segment, 3°) rejoining and sealing with the original segment. Chromosomes numbered according to original karyotype descriptions (see Fig. 1). Bar = 5 µm.

Comparison of the G-banded karyotypes of the two *Cavia* species included in this study revealed that most of the autosomal pairs and the X chromosomes showed total G band correspondence, suggesting that these chromosomes constitute large syntenic blocks present in the common ancestor of both species and conserved over time. The differences in morphology and the partial G band correspondences detected between four chromosomal pairs of these two species, suggest that the four submetacentric chromosomes present only in the *Cavia tschudii* karyotype would have suffered pericentric inversions originating the four subtelocentric chromosomes of *Cavia porcellus* (Fig. 6, Table 1).

*Cavia porcellus* would be the domestic successor of *Cavia tschudii* from which it would have originated more than 4000 and possibly 7000 years ago (Wing 1986) by a process of domestication and artificial selection in the *Cavia tschudii* populations which inhabit southern Peru and northern Chile (Spotorno et al. 2007, Dunnum and Salazar-Bravo 2010). While being domesticated, those populations must have been small in size and with only a few individuals participating as parents in the reproductive process, which over many generations would have produced high levels of endogamy. These characteristics would have facilitated the fixation of the pericentric inversions which must have emerged spontaneously and frequently in the populations. Specifically, the heterozygotes for the pericentric inversions would have decreased their fertility since their gametes would be unbalanced as a result of crossing-over in the inverted segment (Coyne et al. 1993, King 1993). As a consequence, gene flow between the original homozygotes and the homozygotes for the inversion would have been reduced, originating genetic divergence between the two chromosomal forms.

Nevertheless, it has been demonstrated recently that the fertility of the inversion carriers is not always reduced (Muss and Schwanitz 2007). In some cases and depending on the size, genetic content, and chromosomal location of the inversion, the chromosomal inverted region pairs non-homologously with its normal partner forming a straight bivalent which does not present any loop, so causing crossing-over suppression (Torgasheva and Borodin 2010). If that is the case, the absence or reduced recombination between the inverted and non inverted genomic regions in the *Cavia* pericentric inversions, would be the cause of genetic divergence accumulation and reduction of gene flow between the two chromosomal forms, as it was proposed as a general model of speciation by several authors (Noor et al. 2001, Navarro and Barton 2003, Hoffman and Rieseberg 2008).

A critical assessment of reproductive isolation in crosses between *Cavia* species as previously reported, confronts the appropriate identification of specimens, the reliability of the taxonomy at the time, and the nature of the differences eventually found. For instance, crosses between *Cavia porcellus* and individuals from Arequipa, Perú identified as *Cavia cutleri* Bennett, 1836, which correspond to the original description of *Cavia cutleri* based on a single specimen from Ica, Peru (see Weir 1974), produced fertile offspring according to Castle (1916). By contrast, other wild specimens from Ica, Peru that also received the name *Cavia cutleri* by Tschudi in 1849, were finally renamed as *Cavia tschudii* by Fitzinger in 1867 (see Weir 1974); the latter is now the usually accepted name for the wild montane guinea pig (Woods and Kilpatrick 2005). Therefore, *Cavia cutleri* Bennet, 1838 is now considered a synonym of *Cavia porcellus* (Woods and Kilpatrick 2005). If Castle in fact crossed *Cavia porcellus* laboratory animals with *Cavia cutleri* Bennet (= *Cavia porcellus*) specimens, in reality he might be doing intraspecific crosses, and the fertility of the descendants would be an expected result. In any case, the assignation of the individuals from Arequipa to *Cavia cutleri* Bennet was not well documented in that study, since it was based only on the smaller body size of those individuals with respect to domestic *Cavia porcellus* (Castle 1916). Moreover, he did not indicated the mating times taken by the crosses, neither the number of pairs in which crosses were attempted, reporting only that a large number of descendants were obtained (n = 107), as many as those obtained in crosses within each form (n = 108). In sum, if the chromosomal and nucleolar differences we are reporting here in molecularly identified specimens of *Cavia tschudii* and *Cavia porcellus* were also found in other populations, we predict that their eventual hybrids will show some degree of genomic incompatibility.

Reproduction of wild mammal species in captivity is a difficult and not always successful task. It is even more difficult to obtain descendants from crosses between different chromosomal races or species in the laboratory (Walker et al. 1984, 1999, Hauffe and Searle 1998, Castiglia and Capanna 2000, Franchini et al. 2008, Nunes et al. 2011). In crosses between phyllotine rodent species, we reported previously a decrease in the proportion of pairs with births and in the litter’s size together with an increase in the time between mating and birth, compared to those registered for the intraspecific crosses (Walker et al. 1984, 1999). Although we repeatedly tried to cross our specimens of *Cavia tschudii* with *Cavia porcellus* in our laboratory, we have had no success yet.

If the analysis of the crosses realized between chromosomal races of *Mus* and *Sorex* rodents (Hauffe and Searle 1998, Castiglia and Capanna 2000, Franchini et al. 2008, Nunes et al. 2011) would have only considered the number of descendants obtained, it would not have been possible to reach conclusions about the fertility level of those hybrids. To estimate fertility, specific reproductive aspects must be studied, such as the success obtained in crosses between the parental forms and some hybrid characters, i.e.: the normality of their meiotic process, the histology of their gonads, the cell composition of their germinal line and the chromosomal constitution of the gametes that eventually they produce. In consequence, to evaluate the fertility level of eventual *Cavia tschudii* × *Cavia porcellus* hybrids, the reproductive characters just mentioned above must be analyzed in the descendants of crosses between individuals of the parental species taxonomically well identified. Specifically, the fertility of the heterozygotes for the pericentric inversions described here should be further investigated to evaluate the contribution of those chromosomal changes to the divergence of the two *Cavia* species.
